# Phosphorylation of the AMPAR-TARP Complex in Synaptic Plasticity

**DOI:** 10.3390/proteomes6040040

**Published:** 2018-10-08

**Authors:** Joongkyu Park

**Affiliations:** 1 Department of Pharmacology, Wayne State University School of Medicine, Detroit, MI 48201, USA; joongkyu.park@wayne.edu; Tel.: +1-303-577-1580; 2 Department of Neurology, Wayne State University School of Medicine, Detroit, MI 48201, USA

**Keywords:** phosphorylation, AMPA receptor complex, transmembrane AMPA receptor regulatory protein, synaptic plasticity

## Abstract

Synaptic plasticity has been considered a key mechanism underlying many brain functions including learning, memory, and drug addiction. An increase or decrease in synaptic activity of the α-amino-3-hydroxy-5-methyl-4-isoxazolepropionic acid receptor (AMPAR) complex mediates the phenomena as shown in the cellular models of synaptic plasticity, long-term potentiation (LTP), and depression (LTD). In particular, protein phosphorylation shares the spotlight in expressing the synaptic plasticity. This review summarizes the studies on phosphorylation of the AMPAR pore-forming subunits and auxiliary proteins including transmembrane AMPA receptor regulatory proteins (TARPs) and discusses its role in synaptic plasticity.

## 1. Introduction

Animal behavior is dynamic. One of the essential features of brain function is the ability to be dynamic in order to express various behaviors. Selective strengthening and weakening of synaptic transmission have been modeled as a critical mechanism for many brain functions including learning, memory, and drug addiction. Long-term potentiation (LTP) and depression (LTD) are well-characterized models of synaptic plasticity, and they can be regulated by changes at presynaptic (e.g., changes in the release of neurotransmitters) and postsynaptic (e.g., changes in the number and properties of neurotransmitter receptors) sites. Importance of the α-amino-3-hydroxy-5-methyl-4-isoxazolepropionic acid receptor (AMPAR) complex has emerged notably in LTP and LTD. In particular, protein phosphorylation is well known to play a pivotal role in the expression of synaptic plasticity, for example, Ca^2+^/CaM-dependent protein kinase II (CaMKII) in hippocampal LTP [1,2,3]. This review gives an overview of the studies on phosphorylation of the AMPAR pore-forming subunits and auxiliary proteins including transmembrane AMPA receptor regulatory proteins (TARPs) and discusses its role in those plastic cellular phenomena.

## 2. AMPAR Complex

AMPARs are predominantly distributed at excitatory synapses and mediate the majority of fast transmission. The AMPAR complex consists of four pore-forming subunits (GluA1–4) and auxiliary proteins including TARP, cornichons-like (CNIH), and cysteine-knot AMPAR modulating protein (CKAMP)/Shisa family in the brain [4,5,6,7,8,9]. Knockout mice of GluA1, TARPγ-8, or CNIH-2/-3 show a substantial reduction in hippocampal LTP [10,11,12], and GluA2 knockout leads to LTD impairment in cultured cerebellar neurons and anterior cingulated cortex slices [13,14], highlighting their significance for synaptic plasticity.

## 3. Phosphorylation of the Pore-Forming Subunits: GluA1

**Figure 1:**
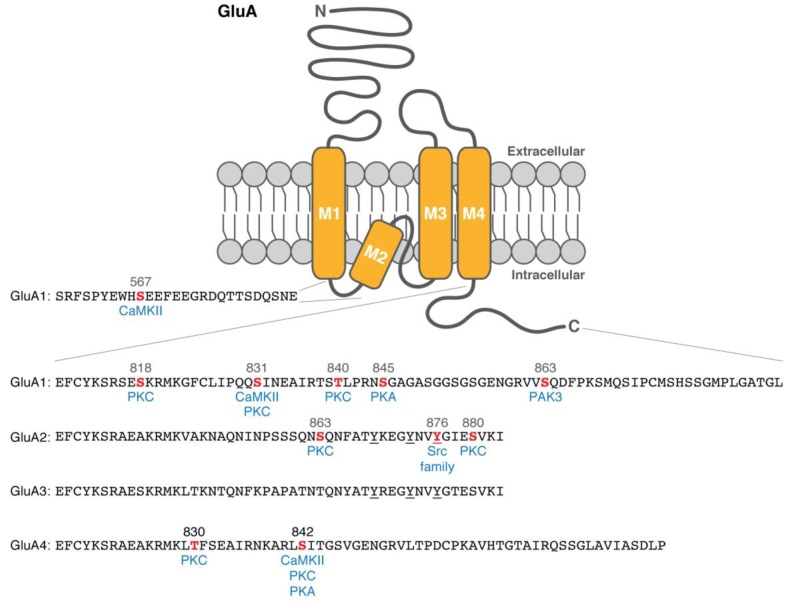
Schematic illustration of α-amino-3-hydroxy-5-methyl-4-isoxazolepropionic acid receptor (AMPAR) subunit structure and phosphorylation sites in the intracellular loop 1 and C-terminal region (referred to [15]). The identified phosphorylation sites are shown in red, and the protein kinases are listed below the sites in blue. The conserved three tyrosine residues on GluA2 and GluA3 subunits are underlined. M1–4 indicates transmembrane domains. CaMKII = Ca^2+^/CaM-dependent protein kinase II; PKC = protein kinase C; PKA3 = cAMP-dependent protein kinase; PAK = p21-activated kinase-3.

To elucidate how phosphorylation regulates synaptic plasticity, extensive research has been conducted to identify phosphorylation sites on the pore-forming subunits [15]. Based on the revised topology [16], the C-terminal intracellular region of the GluA1 subunit has emerged as a potential phosphorylation target (Figure 1).

A serine residue (Ser) at 831 in the intracellular region (Ser831) is one of the most attractive phosphorylation sites of the GluA1 subunit. In vitro phosphorylation assays reveal that Ser831 can be directly phosphorylated by CaMKII [17] or protein kinase C (PKC) [18]. Ser831 phosphorylation is also shown on transiently expressed GluA1 in a heterologous cell line (a quail-origin fibroblast, QT6) co-transfected with a constitutively active form of CaMKII [19] and in a PKC- and cAMP-dependent protein kinase (PKA)-activating condition of human embryonic kidney 293 (HEK293) cells (treated with phorbol 12-myristate 13-acetate, forskolin, and 3-isobutyl-1-methylxanthine) [18], respectively. Therefore, this single site can be a shared target that can be phosphorylated by two different kinases: CaMKII and PKC. Functional studies with whole-cell recording or outside-out membrane patches reveal that Ser831 phosphorylation is critical for CaMKII- or PKC-induced AMPAR potentiation and channel conductance enhancement in heterologous systems [17,20,21].

A serine residue at 845 (Ser845) is another phosphorylation target site that has been characterized well. In vitro phosphorylation assay with purified PKA identifies direct phosphorylation at Ser845 [18]. In heterologous cells, PKA activation (by forskolin and 3-isobutyl-1-methylxanthine) induces Ser845 phosphorylation on transiently expressed GluA1 [18,19]. Recording of whole-cell patches or single-channel currents with PKA infusion reveals that Ser845 is necessary for PKA-induced AMPAR potentiation and enhancement of channel open probability [18,22]. The positive effects of Ser831 and Ser845 phosphorylation on AMPAR potentiation and channel conductance may contribute to synaptic plasticity upon stimulation.

In ex vivo slices, phosphorylation at Ser831 and Ser845 of GluA1 highly correlates with LTP and LTD. To describe their phosphorylation states, phosphorylation site-specific antibodies have been developed and validated by activation of PKC (by phorbol dibutyrate) and PKA (by forskolin and 3-isobutyl-1-methylxanthine) in rat hippocampal slices [19]. Theta burst stimulation (TBS)-induced LTP increases Ser831 phosphorylation (but not at Ser845) in rat hippocampal slices, and the subsequent low-frequency stimulation (LFS)-induced depotentiation decreases the phosphorylation [23]. This is consistent with the notion that Ser831 residue of GluA1 subunit can be phosphorylated by CaMKII, which is required for LTP [3,17,24]. In contrast, Ser845 phosphorylation (not at Ser831) reduces in the LFS-induced LTD condition of rat hippocampal slices [23].

PKC is known to phosphorylate other sites on the GluA1 subunit. Autoradiograph and phosphorylation site-specific antibody combined with purified C-terminal mutants of GluA1 reveal that PKC (but not CaMKII) can directly phosphorylate Ser818 in vitro, and Ser818 phosphorylation increases upon chemical LTP and TBS [25]. Thr840 is another PKC target site that was uncovered by in vitro phosphorylation assays, a phosphorylation site-specific antibody, and PKC activation (by phorbol ester) or inhibition (by Gö6976 or chelerythrine) in hippocampal slices [26,27]. Interestingly, Thr840 phosphorylation inhibits a PKA-mediated increase in Ser845 phosphorylation and subsequent AMPAR potentiation whereas a phospho-mimetic aspartate mutation at Ser845 (S845D) inhibits PKC-mediated Thr840 phosphorylation in vitro and in hippocampal slices [27]. This suggests that GluA1 phosphorylation may regulate AMPAR channel properties dynamically depending on upstream kinase signaling pathways. This idea that the GluA1 subunit has a hyper-regulatory domain with multiple phosphorylation sites is also supported by a study that single or multiple aspartate mutations at Ser818, Thr840, and Ser831 residues enhance the weighted mean channel conductance [28]. In addition, surface expression and synaptic trafficking of AMPARs can be regulated by p21-activated kinase-3 (PAK3)-mediated Ser863 phosphorylation [29] and CaMKII-mediated phosphorylation of Ser567 residue in the loop 1 of GluA1 subunits [30]. This information is listed in Table 1.

## 4. Significance of GluA1 Phosphorylation for Synaptic Plasticity: Knock-In Mouse Studies

The phosphorylation studies using heterologous cell systems and overexpression of GluA1 mutants have provided us with valuable mechanistic information. However, it has to be validated in more physiological conditions such as targeted knock-in mouse models (Table 2). A ‘Penta’ phosphomutant mouse line is generated with alanine mutations at Ser831, Thr838, Ser839, Thr840, and Ser845 [26]. The ‘Penta’ phosphomutant mice show a reduction in LTP and LTD compared to that of wild-type mice at adult (~3-month-old) but not young (3–4-week-old) stage [26], suggesting that some or all of those five phosphorylation sites are necessary for synaptic plasticity and there are different mechanisms involved in those phenomena depending on age. The reduced LTP and LTD in adult mice are also shown in a ‘Double’ phosphomutant mouse line that lacks phosphorylation at Ser831 and Ser845 residues, suggesting that the other three sites including Thr840 may not contribute additionally to synaptic plasticity [43]. Interestingly, a single knock-in mouse that has an alanine mutation at Ser831 residue exhibits intact LTP and LTD at adult and young stages [44]. This may suggest that there are other CaMKII substrates that contribute to LTP more than Ser831 phosphorylation of GluA1 subunits because disrupting CaMKII shows impairment in LTP in a knockout mouse or a knock-in mouse of a mutant CaMKII [3,24]. One of the possible candidates for CaMKII substrates could be Ser567 residue in the loop 1 of GluA1 subunits as it is shown to be involved in the synaptic targeting of AMPARs [30], but Ser567 phosphorylation has not been validated yet by a knock-in study. On the other hand, a single knock-in mouse that lacks Ser845 phosphorylation displays an abolished LTD at adult and young stages [44], consistent with the previous finding that Ser845 phosphorylation correlates with LFS-induced LTD condition in rat hippocampal slices [23].

## 5. Phosphorylation of the Pore-Forming Subunits: GluA2 and GluA3

Unlike GluA1, no CaMKII phosphorylation is detected on transiently expressed GluA2 in HEK293 cells [17]. However, the C-terminal intracellular region of the GluA2 subunit serves phosphorylation substrate sites (Table 1) despite the limited homology to the GluA1’s C-terminal region (Figure 1). In vitro phosphorylation and phosphopeptide mapping reveal that PKC can directly phosphorylate GluA2 subunits at Ser863 and Ser880 [31,33]. Phosphorylation site-specific antibodies also show that Ser863 and Ser880 residues are phosphorylated on transiently expressed GluA2 in a PKC-activating condition of HEK293 cells (treated by phorbol 12-myristate 13-acetate) [31,33]. The Ser863 phosphorylation likely exists in vivo brains as shown by an immunoblot of rat brain homogenates using the phosphorylation site-specific antibody [31].

LTD is abolished in cultured cerebellar Purkinje cells from GluA2 knockout mice, and the abolished LTD can be rescued by transient expression of the wild-type GluA2 subunit [13]. However, expression of a mutant form of GluA2 that lacks Ser880 phosphorylation fails to restore LTD whereas expression of its phospho-mimetic form with a glutamate mutation at Ser880 residue occludes LTD, suggesting the importance of Ser880 phosphorylation in cerebellar LTD [13].

The C-terminal ends of GluA2 and GluA3 subunits uniquely have three conserved tyrosine residues (Figure 1). Immunoblots with anti-phosphotyrosine (PY20) antibody of immunoprecipitated GluA2 or GluA3 from mouse brains show their tyrosine phosphorylation [32]. Tyrosine phosphorylation-specific antibody against the C-terminal part of GluA2 subunits and site-specific mutants further identify that a Src family protein tyrosine kinase Lyn phosphorylates Tyr876 of GluA2 subunits [32]. The phosphorylation at the C-terminal end of GluA2 subunits (i.e., Tyr876 and Ser880 adjacent to its PDZ-binding motif) negatively regulates GluA2 binding to glutamate receptor-interacting protein (GRIP) [32,33], which is a synaptic PDZ domain-containing protein [45]. The C-terminal tyrosine phosphorylation on GluA2 subunits (including Tyr876) is required for AMPA-, N-methyl-D-aspartate (NMDA)-, and insulin-induced internalization of GluA2 subunits in cultured cortical neurons [32,34]. LFS-induced LTD condition correlates with an increase in tyrosine phosphorylation of GluA2 subunits in homogenates from the stimulated rat hippocampal slices [34]. Also, the C-terminal peptide of wild-type GluA2 (but not a mutant with alanine substitution at Tyr869, Tyr873, and Tyr876) in an intracellular recording solution interferes with LFS-induced LTD, suggesting that tyrosine phosphorylation of GluA2 subunits is required for LFS-induced hippocampal LTD [34].

## 6. Phosphorylation of the Pore-Forming Subunits: GluA4

Transiently expressed GluA4 subunits are phosphorylated in a PKC-activating (by phorbol 12-myristate 13-acetate) or PKA-activating (by forskolin) condition of HEK293T cells [35]. Phosphopeptide mapping identifies Ser842 residue as the major phosphorylation site in the C-terminal intracellular region of GluA4 subunits, which can be phosphorylated by CaMKII, PKC, and PKA in vitro and in a PKA-activating condition of HEK293T cells [35] (Table 1). Also, PKC can phosphorylate Thr830 residue of GluA4 [35].

PKA activity is necessary and sufficient for synaptic incorporation of GluA4 subunits in hippocampal slices [36]. The PKA activation (by forskolin and 3-isobutyl-1-methylxanthine) leads to AMPAR potentiation and an increase in Ser842 phosphorylation of GluA4 subunits in hippocampal slices [36].

## 7. TARP Phosphorylation and Its Roles in LTP

**Figure 2:**
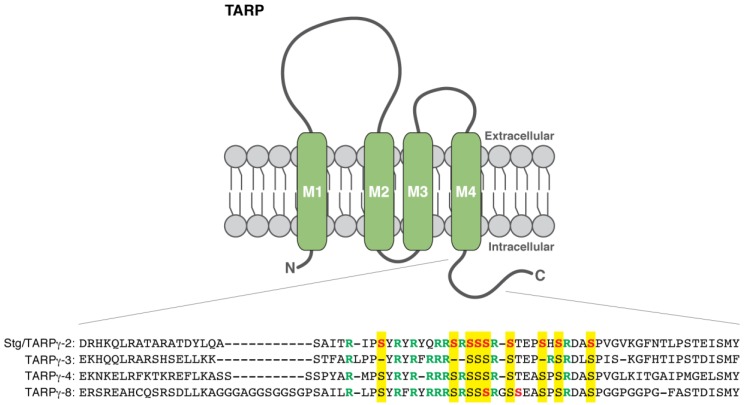
Schematic illustration of TARP isoform structure and phosphorylation sites in the part of the C-terminal cytoplasmic tail of each isoform (referred to [37]). The identified phosphorylation sites are shown in red, and adjacent arginine residues are indicated in green. The conserved serine residues of stargazin/TARPγ-2 phosphorylation sites are highlighted in yellow. M1–4 indicates transmembrane domains.

AMPARs exist in the brain as a protein complex with auxiliary proteins (e.g., TARP, CNIH, and CKAMP/Shisa) [4,9,46,47,48]. Stargazin/TARPγ-2 is firstly focused, as its mutant mice (termed *stargazer*) show a loss of AMPAR-mediated transmission in cerebellar granule cells [49,50]. The TARP family comprises two classes, type I (stargazin/TARPγ-2, γ-3, γ-4, and γ-8) and type II (TARPγ-5 and γ-7) [9,51]. TARPs have four transmembrane domains (Figure 2) and regulate trafficking and channel properties of AMPARs [9,52,53,54]. TARP shows differential expression patterns in adult rodent brains, for example, stargazin/TARPγ-2 is the dominant isoform of TARPs in the cerebellum whereas TARPγ-8 is highly enriched in the hippocampus [51], suggesting distinct roles of each member in different brain regions.

Phosphorylation of stargazin/TARPγ-2 was firstly described. Both extra-synaptic (Triton X-100-soluble) and post-synaptic density (Triton X-100-insoluble) fractions show phosphorylation of stargazin/TARPγ-2 [37]. Intriguingly, the post-synaptic density fraction dominantly displays the highest degree of stargazin/TARPγ-2 phosphorylation whereas the extra-synaptic fraction has multiple phosphorylation bands with variable degrees, suggesting that synaptic stargazin/TARPγ-2 is preferentially phosphorylated [37]. The phosphorylation sites of stargazin/TARPγ-2 are identified as nine serine residues at 228, 237, 239, 240, 241, 243, 247, 249, and 253 in the C-terminal cytoplasmic tail (mouse stargazin/TARPγ-2) by phosphopeptide mapping with primary cortical neurons and transfected Chinese hamster ovary (CHO) cells [37] (Table 1). CaMKII and PKC can be the kinases for stargazin/TARPγ-2 phosphorylation as shown by in vitro phosphorylation assays and inhibitor studies [37]. A stargazin/TARPγ-2 phospho-mimetic knock-in mouse line that has aspartate mutations at all those nine serine residues (S9D) (Stargazin^SD^) shows an increase in synaptic AMPAR activity in cerebellar mossy fiber-granule cell synapses [38], consistent with the notion that phosphorylated forms of stargazin/TARPγ-2 are dominant in the post-synaptic density fraction [37]. In hippocampal slice cultures, LTP is occluded by expression of a phospho-mimetic form of stargazin/TARPγ-2 (S9D) and prevented by expression of a phospho-deficient stargazin/TARPγ-2 that has alanine mutations (S9A) [37]. Also, Thr321 of stargazin/TARPγ-2 can be phosphorylated by PKA, extracellular signal-regulated protein kinase 2, and p38 mitogen-activated protein kinase in vitro [40].

Phosphorylation of TARPγ-8 by CaMKII is shown by in vitro phosphorylation assays [41]. The nine phosphorylation sites of stargazin/TARPγ-2 are highly conserved in all four TARP isoforms including TARPγ-8 (e.g., serine at 264, 273, 275, 276, 277, 280, 284, 286, and 290 of mouse TARPγ-8), and TARPγ-8 uniquely has one more serine residue at 281 [37,41] (Figure 2). In vitro phosphorylation assay and radio-Edman sequencing identify Ser277 and Ser281 as CaMKII phosphorylation sites on TARPγ-8 [41] (Table 1). A TARPγ-8 knock-in mouse line containing alanine mutations at Ser277 and Ser281 residues (TARPγ-8^Cm^) shows a substantial reduction in hippocampal LTP, suggesting that CaMKII phosphorylation of TARPγ-8 at these two sites is required for LTP [41] (Table 2). The possible contribution of stargazin/TARPγ-2 phosphorylation, TARPγ-3, and TARPγ-4 to hippocampal LTP may be excluded because hippocampal LTP is intact in both a stargazin/TARPγ-2 knock-in mouse line that lacks the nine phosphorylation sites (Stargazin^SA^) and a triple mutant mouse with Stargazin^SA^ knock-in plus knockout of TARPγ-3 and TARPγ-4 [41]. Previously, TARPγ-8 knockout mice exhibited a substantial reduction in LTP, but not LTD, as well as altered expression of AMPAR subunits (i.e., GluA1 and GluA2) [11]. Possible secondary effect of the altered protein expression on the LTP impairment can be ruled out because the TARPγ-8^Cm^ knock-in mice with S277A and S281A show no obvious differences in AMPAR expression compared to wild-type mice [41]. In addition, overexpression of a TARPγ-8 phospho-mimetic form with aspartate mutations at Ser277 and Ser281 is sufficient to enhance synaptic AMPAR activity in the hippocampus [41].

The molecular mechanism for how TARP phosphorylation regulates synaptic AMPAR activity is one of the most interesting topics in synaptic plasticity. In the C-terminal cytoplasmic tail of TARPs, the serine sites are adjacent to many arginine residues (e.g., arginine residues at 225, 230, 232, 235, 236, 238, 242, and 250 of mouse stargazin/TARPγ-2) (Figure 2). The positive charges from these arginine residues of stargazin/TARPγ-2 can directly bind to negatively charged lipids in vitro, for example, phosphatidic acid, phosphatidylinositol-4-phosphate (PIP), phosphatidylinositol-4,5-bisphosphate (PIP_2_), and phosphatidylinositol-3,4-5-triphosphate (PIP_3_) [38]. Phospho-mimetic S9D mutations (aspartate substitution at nine serine residues of stargazin/TARPγ-2) disrupt the electrostatic interaction between the cytoplasmic domain of stargazin/TARPγ-2 and the negatively charged lipids [38]. Consistently, the charge-dependent dissociation of the TARP cytoplasmic domain from the plasma membrane occurs in cultured hippocampal neurons [39]. Fluorescence lifetime imaging microscopy reveals that the phospho-mimetic S9D mutant of stargazin/TARPγ-2 (GFP-tagged right after the arginine/serine-rich domain) exhibits a longer GFP lifetime than wild-type stargazin/TARPγ-2 due to being further from the plasma membrane (stained by a plasma membrane marker R18; octadecyl rhodamine B chloride), suggesting that the C-terminus of the phospho-mimetic form of stargazin/TARPγ-2 extends further into the cytoplasm than wild-type [39]. As discussed earlier, highly phosphorylated forms of stargazin/TARPγ-2 are dominant in the post-synaptic density fraction [37], and the Stargazin^SD^ knock-in mice show an increase in synaptic AMPAR activity [38]. Taken together, TARP phosphorylation may disrupt the membrane binding of the C-terminal cytoplasmic tail to trigger a synaptic enhancement of AMPAR activity.

Since all TARP isoforms commonly have the eight to nine arginine residues adjacent to the phosphorylation sites (Figure 2), it would not be surprising that other TARP isoforms behave in a similar way to stargazin/TARPγ-2 in terms of the binding to/dissociation from membranes. For example, it is possible that CaMKII phosphorylation at Ser277 and Ser281 of TARPγ-8 may lead to dissociation of the cytoplasmic tail from the plasma membrane to enhance synaptic AMPAR activity in the hippocampus as does stargazin/TARPγ-2 in cerebellar granule cells. This model is further supported by a study with a TARPγ-8 knock-in mouse line (TARPγ-8^Δ4^) that lacks the last four amino acids, a PDZ-binding motif. The interaction between PDZ-binding motifs of TARPs and membrane-associated guanylate kinase family proteins (e.g., PSD-95) is proposed to stabilize AMPAR-TARP complexes at synapses. Consistent with this idea, TARPγ-8^Δ4^ knock-in mice exhibit ~30% reduction in basal AMPAR transmission in the hippocampus [55]. However, hippocampal LTP is intact in the TARPγ-8^Δ4^ knock-in mice [55], suggesting that PDZ binding of TARPγ-8 is not necessary for LTP expression.

## 8. Phosphorylation of Other Auxiliary Proteins of AMPAR

Accumulating reports have identified more auxiliary proteins of the AMPAR complex, such as CNIH, germ cell-specific gene 1-like protein (GSG1-L), and CKAMP/Shisa [9,46,47,48]. CNIH-2/-3 are identified by proteomic analysis of native AMPAR complexes from rat brains [56]. CNIHs regulate surface expression and channel properties of AMPARs in heterologous cells and mouse brain slices [12,56,57]. GSG1-L also modulates AMPAR trafficking and desensitization in heterologous cells [58,59]. However, phosphorylation of CNIHs and GSG1-L remains unknown.

Among the binding proteins to AMPARs [47,58], CKAMP44/Shisa9 is known to be phosphorylated by PKC and protein interacting with C kinase 1 (PICK1) [42]. In vitro phosphorylation assay and Phos-tag polyacrylamide gel electrophoresis reveal that CKAMP44/Shisa9 can be phosphorylated by PKC in vitro and in a PKC-activating condition of COS-7 cells (treated by phorbol 12-myristate 13-acetate) [42]. However, the phosphorylation sites and their involvement in synaptic plasticity are unknown.

## 9. Closing Remarks

Phosphorylation of the pore-forming subunits and auxiliary proteins of the AMPAR complex has been extensively studied. The broad spectrum of biochemical approaches such as in vitro phosphorylation assay, phosphopeptide mapping, phosphorylation site-specific antibodies, and Phos-tag polyacrylamide gel electrophoresis has allowed us to identify the actual phosphorylation sites and to describe their states in synaptic plasticity. Combined with the biochemical approaches, in vivo and ex vivo studies including genetically modified mice and electrophysiological analyses have found compelling phenomena and their molecular and cellular mechanisms. However, many questions remain in the field of synaptic plasticity, in particular, in regards to proteomics. Although the native AMPAR complex constituents were recently profiled by proteomics [58], phosphoproteomics of the native complex has not been reported yet. Very little is known to date regarding phosphorylation of many AMPAR complex constituents including TARPs, CNIHs, GSG1-L, and CKAMPs. Also, roles of the previously identified phosphorylation sites of the complex in synaptic plasticity need to be investigated further in more physiological conditions (e.g., targeted knock-in mice). Accumulating knowledge from the phosphorylation studies of the native AMPAR complex will provide us with more insight into the activity-dependent dynamic changes at synapses that underlie various animal behaviors. Although this review is only limited to AMPAR complex proteins, other synaptic proteins including neurotransmitter receptors and scaffolding proteins may serve as phosphorylation substrates that contribute to synaptic plasticity.

## Figures and Tables

**Table 1:** Phosphorylation of AMPAR pore-forming subunits and auxiliary proteins and their involvement in synaptic plasticity. LTP = long-term potentiation; LTD = long-term depression; TBS = theta burst stimulation; KO = knockout; TARP = transmembrane AMPA receptor regulatory proteins; CKAMP = cysteine-knot AMPAR modulating protein; GRIP = glutamate receptor-interacting protein; ERK2 = extracellular signal-regulated protein kinase 2; p38 MAPK = p38 mitogen-activated protein kinase.

Protein	Target Site	Kinase	Identification	Effect on the AMPAR Complex	Involvement in Synaptic Plasticity
GluA1	Ser567	CaMKII	In vitro; Phospho-specific antibody with rat hippocampal lysate [30]	Regulation of synaptic trafficking [30]	
	Ser818	PKC	In vitro; PKC activation of heterologous cells; Phospho-specific antibody with rat cortical lysate [25]	Enhancement of the weighted mean channel conductance [28]	A correlational increase upon chemical LTP and TBS [25]
	Ser831	CaMKII	In vitro [17]; Co-expression of a constitutively active CaMKII in heterologous cells [19]; Phospho-specific antibody with rat hippocampal lysate [19,23]	Potentiation of AMPAR current [17]; Enhancement of channel conductance [20,21]	A correlational increase upon LTP [23]
		PKC	In vitro; PKC- and PKA-activation of heterologous cells [18]		
	Thr840	PKC	In vitro [26,27]; Phosphopeptide mapping of hippocampal slices [26]; Phospho-specific antibody with mouse hippocampal lysate [26,27]	Inhibition of PKA-induced AMPAR potentiation [27]; Enhancement of the weighted mean channel conductance [28]	A correlational change upon PKC activity [26,27]
	Ser845	PKA	In vitro [18]; PKA activation of heterologous cells [18,19]; Phospho-specific antibody with rat hippocampal lysate [19,23]	Potentiation of AMPAR current [18]; Enhancement of channel opening probability [22]	A correlational decrease upon LTD [23]
	Ser863	PAK3	In vitro; Co-expression in heterologous cells; Phospho-specific antibody with cortical lysate [29]	Regulation of surface expression [29]	
GluA2	Ser863	PKC	In vitro; PKC activation of heterologous cells; Phospho-specific antibody with cortical lysate [31]		
	Tyr876	Lyn	Co-expression in heterologous cells [32]	Regulation of GRIP binding [32,33]	
	Tyr869, Tyr873, Tyr876			Internalization of GluA2 subunits [32,34]	A correlational increase upon LTD [34]
	Ser880	PKC	In vitro; PKC activation of heterologous cells [31,33]	Regulation of GRIP binding [33]	Contribution to LTD in GluA2 KO cerebellar Purkinje cell cultures [13]
GluA4	Thr830	PKC	In vitro [35]		
	Ser842	CaMKII	In vitro [35]	Synaptic incorporation of GluA4 subunits [36]	A correlational increase upon PKA activation [36]
		PKC	In vitro [35]		
		PKA	In vitro; PKC activation of heterologous cells [35]		
Stargazin/TARPγ-2	Ser228, Ser237, Ser239, Ser240, Ser241, Ser243, Ser247, Ser249, Ser253	CaMKII, PKC	Phosphopeptide mapping of heterologous cells and cortical neurons [37]	Dissociation of the cytoplasmic domain from the plasma membrane [38,39]	Contribution to LTP in hippocampal slice culture [37]
	Thr321	PKA, ERK2, p38 MAPK	In vitro [40]	Regulation of PSD-95 binding [40]	
TARPγ-8	Ser277, Ser281	CaMKII	In vitro; Radio-Edman sequencing [41]	Enhancement of synaptic AMPAR activity [41]	A correlational increase upon chemical LTP [41]
CKAMP44/Shisa9	N/A	PKC	In vitro; PKC activation of heterologous cells [42]		

**Table 2:** Targeted knock-in mouse studies of AMPAR and TARP phosphorylation sites.

Protein	Phosphorylation Site (Mutated to Alanine Residues)	Effect on Synaptic Plasticity
GluA1	Ser831, Thr838, Ser839, Thr840, and Ser845	A reduction in LTP and LTD at adult stage [26]
	Ser831 and Ser845	A reduction in LTP and LTD at adult stage [43]
	Ser831	Normal LTP and LTD [44]
	Ser845	Abolished LTD [44]
Stargazin/TARPγ-2	Ser228, Ser237, Ser239, Ser240, Ser241, Ser243, Ser247, Ser249, and Ser253	Normal LTP [41]
TARPγ-8	Ser277 and Ser281	A reduction in LTP [41]

## References

[1] Malenka R.C., Kauer J.A., Perkel D.J., Mauk M.D., Kelly P.T., Nicoll R.A., Waxham M.N. (1989). An essential role for postsynaptic calmodulin and protein kinase activity in long-term potentiation. Nature.

[2] Malinow R., Schulman H., Tsien R.W. (1989). Inhibition of postsynaptic PKC or CaMKII blocks induction but not expression of LTP. Science.

[3] Silva A.J., Stevens C.F., Tonegawa S., Wang Y. (1992). Deficient hippocampal long-term potentiation in alpha-calcium-calmodulin kinase II mutant mice. Science.

[4] Nicoll R.A., Tomita S., Bredt D.S. (2006). Auxiliary subunits assist AMPA-type glutamate receptors. Science.

[5] Jackson A.C., Nicoll R.A. (2011). The expanding social network of ionotropic glutamate receptors: TARPs and other transmembrane auxiliary subunits. Neuron.

[6] Greger I.H., Watson J.F., Cull-Candy S.G. (2017). Structural and functional architecture of AMPA-type glutamate receptors and their auxiliary proteins. Neuron.

[7] Twomey E.C., Yelshanskaya M.V., Grassucci R.A., Frank J., Sobolevsky A.I. (2016). Elucidation of AMPA receptor-stargazin complexes by cryo-electron microscopy. Science.

[8] Chen S., Zhao Y., Wang Y., Shekhar M., Tajkhorshid E., Gouaux E. (2017). Activation and desensitization mechanism of AMPA receptor-TARP complex by cryo-EM. Cell.

[9] Yan D., Tomita S. (2012). Defined criteria for auxiliary subunits of glutamate receptors. J. Physiol..

[10] Zamanillo D., Sprengel R., Hvalby O., Jensen V., Burnashev N., Rozov A., Kaiser K.M., Köster H.J., Borchardt T., Worley P. (1999). Importance of AMPA receptors for hippocampal synaptic plasticity but not for spatial learning. Science.

[11] Rouach N., Byrd K., Petralia R.S., Elias G.M., Adesnik H., Tomita S., Karimzadegan S., Kealey C., Bredt D.S., Nicoll R.A. (2005). TARP gamma-8 controls hippocampal AMPA receptor number, distribution and synaptic plasticity. Nat. Neurosci..

[12] Herring B.E., Shi Y., Suh Y.H., Zheng C.Y., Blankenship S.M., Roche K.W., Nicoll R.A. (2013). Cornichon proteins determine the subunit composition of synaptic AMPA receptors. Neuron.

[13] Chung H.J., Steinberg J.P., Huganir R.L., Linden D.J. (2003). Requirement of AMPA receptor GluR2 phosphorylation for cerebellar long-term depression. Science.

[14] Toyoda H., Wu L.J., Zhao M.G., Xu H., Jia Z., Zhuo M. (2007). Long-term depression requires postsynaptic AMPA GluR2 receptor in adult mouse cingulate cortex. J. Cell. Physiol..

[15] Wang J.Q., Arora A., Yang L., Parelkar N.K., Zhang G., Liu X., Choe E.S., Mao L. (2005). Phosphorylation of AMPA receptors: mechanisms and synaptic plasticity. Mol. Neurobiol..

[16] Hollmann M., Maron C., Heinemann S. (1994). N-Glycosylation site tagging suggests a three transmembrane domain topology for the glutamate receptor GluR1. Neuron.

[17] Barria A., Derkach V., Soderling T. (1997). Identification of the Ca^2+^/calmodulin-dependent protein kinase II regulatory phosphorylation site in the alpha-amino-3-hydroxyl-5-methyl-4-isoxazole-propionate-type glutamate receptor. J. Biol. Chem..

[18] Roche K.W., O’Brien R.J., Mammen A.L., Bernhardt J., Huganir R.L. (1996). Characterization of multiple phosphorylation sites on the AMPA receptor GluR1 subunit. Neuron.

[19] Mammen A.L., Kameyama K., Roche K.W., Huganir R.L. (1997). Phosphorylation of the alpha-amino-3-hydroxy-5-methylisoxazole4-propionic acid receptor GluR1 subunit by calcium/calmodulin-dependent kinase II. J. Biol. Chem..

[20] Derkach V., Barria A., Soderling T.R. (1999). Ca^2+^/calmodulin-kinase II enhances channel conductance of alpha-amino-3-hydroxy-5-methyl-4-isoxazolepropionate type glutamate receptors. Proc. Natl. Acad. Sci. USA.

[21] Jenkins M.A., Traynelis S.F. (2012). PKC phosphorylates GluA1-Ser831 to enhance AMPA receptor conductance. Channels.

[22] Banke T.G., Bowie D., Lee H.K., Huganir R.L., Schousboe A., Traynelis S.F. (2000). Control of GluR1 AMPA receptor function by cAMP-dependent protein kinase. J. Neurosci..

[23] Lee H.K., Barbarosie M., Kameyama K., Bear M.F., Huganir R.L. (2000). Regulation of distinct AMPA receptor phosphorylation sites during bidirectional synaptic plasticity. Nature.

[24] Giese K.P., Fedorov N.B., Filipkowski R.K., Silva A.J. (1998). Autophosphorylation at Thr286 of the alpha calcium-calmodulin kinase II in LTP and learning. Science.

[25] Boehm J., Kang M.G., Johnson R.C., Esteban J., Huganir R.L., Malinow R. (2006). Synaptic incorporation of AMPA receptors during LTP is controlled by a PKC phosphorylation site on GluR1. Neuron.

[26] Lee H.K., Takamiya K., Kameyama K., He K., Yu S., Rossetti L., Wilen D., Huganir R.L. (2007). Identification and characterization of a novel phosphorylation site on the GluR1 subunit of AMPA receptors. Mol. Cell. Neurosci..

[27] Gray E.E., Guglietta R., Khakh B.S., O’Dell T.J. (2014). Inhibitory interactions between phosphorylation sites in the C terminus of α-Amino-3-hydroxy-5-methyl-4-isoxazolepropionic acid-type glutamate receptor GluA1 subunits. J. Biol. Chem..

[28] Jenkins M.A., Wells G., Bachman J., Snyder J.P., Jenkins A., Huganir R.L., Oswald R.E., Traynelis S.F. (2014). Regulation of GluA1 α-amino-3-hydroxy-5-methyl-4-isoxazolepropionic acid receptor function by protein kinase C at serine-818 and threonine-840. Mol. Pharmacol..

[29] Hussain N.K., Thomas G.M., Luo J., Huganir R.L. (2015). Regulation of AMPA receptor subunit GluA1 surface expression by PAK3 phosphorylation. Proc. Natl. Acad. Sci. USA.

[30] Lu W., Isozaki K., Roche K.W., Nicoll R.A. (2010). Synaptic targeting of AMPA receptors is regulated by a CaMKII site in the first intracellular loop of GluA1. Proc. Natl. Acad. Sci. USA.

[31] McDonald B.J., Chung H.J., Huganir R.L. (2001). Identification of protein kinase C phosphorylation sites within the AMPA receptor GluR2 subunit. Neuropharmacology.

[32] Hayashi T., Huganir R.L. (2004). Tyrosine phosphorylation and regulation of the AMPA receptor by SRC family tyrosine kinases. J. Neurosci..

[33] Matsuda S., Mikawa S., Hirai H. (1999). Phosphorylation of serine-880 in GluR2 by protein kinase C prevents its C terminus from binding with glutamate receptor-interacting protein. J. Neurochem..

[34] Ahmadian G., Ju W., Liu L., Wyszynski M., Lee S.H., Dunah A.W., Taghibiglou C., Wang Y., Lu J., Wong T.P. (2004). Tyrosine phosphorylation of GluR2 is required for insulin-stimulated AMPA receptor endocytosis and LTD. EMBO J..

[35] Carvalho A.L., Kameyama K., Huganir R.L. (1999). Characterization of phosphorylation sites on the glutamate receptor 4 subunit of the AMPA receptors. J. Neurosci..

[36] Esteban J.A., Shi S.H., Wilson C., Nuriya M., Huganir R.L., Malinow R. (2003). PKA phosphorylation of AMPA receptor subunits controls synaptic trafficking underlying plasticity. Nat. Neurosci..

[37] Tomita S., Stein V., Stocker T.J., Nicoll R.A., Bredt D.S. (2005). Bidirectional synaptic plasticity regulated by phosphorylation of stargazing-like TARPs. Neuron.

[38] Sumioka A., Yan D., Tomita S. (2010). TARP phosphorylation regulates synaptic AMPA receptors through lipid bilayers. Neuron.

[39] Hafner A.S., Penn A.C., Grillo-Bosch D., Retailleau N., Poujol C., Philippat A., Coussen F., Sainlos M., Opazo P., Choquet D. (2015). Lengthening of the Stargazin cytoplasmic tail increases synaptic transmission by promoting interaction to deeper domains of PSD-95. Neuron.

[40] Stein E.L., Chetkovich D.M. (2010). Regulation of stargazin synaptic trafficking by C-terminal PDZ ligand phosphorylation in bidirectional synaptic plasticity. J. Neurochem..

[41] Park J., Chávez A.E., Mineur Y.S., Morimoto-Tomita M., Lutzu S., Kim K.S., Picciotto M.R., Castillo P.E., Tomita S. (2016). CaMKII phosphorylation of TARPγ-8 is a mediator of LTP and learning and memory. Neuron.

[42] Kunde S.A., Rademacher N., Zieger H., Shoichet S.A. (2017). Protein kinase C regulates AMPA receptor auxiliary protein Shisa9/CKAMP44 through interactions with neuronal scaffold PICK1. FEBS Open Bio.

[43] Lee H.K., Takamiya K., Han J.S., Man H., Kim C.H., Rumbaugh G., Yu S., Ding L., He C., Petralia R.S. (2003). Phosphorylation of the AMPA receptor GluR1 subunit is required for synaptic plasticity and retention of spatial memory. Cell.

[44] Lee H.K., Takamiya K., He K., Song L., Huganir R.L. (2010). Specific roles of AMPA receptor subunit GluR1 (GluA1) phosphorylation sites in regulating synaptic plasticity in the CA1 region of hippocampus. J. Neurophysiol..

[45] Dong H., O’Brien R.J., Fung E.T., Lanahan A.A., Worley P.F., Huganir R.L. (1997). GRIP: A synaptic PDZ domain-containing protein that interacts with AMPA receptors. Nature.

[46] Tigaret C., Choquet D. (2009). More AMPAR garnish. Science.

[47] Farrant M., Cull-Candy S.G. (2010). Neuroscience. AMPA receptors—Another twist?. Science.

[48] Haering S.C., Tapken D., Pahl S., Hollmann M. (2014). Auxiliary subunits: shepherding AMPA receptors to the plasma membrane. Membranes.

[49] Hashimoto K., Fukaya M., Qiao X., Sakimura K., Watanabe M., Kano M. (1999). Impairment of AMPA receptor function in cerebellar granule cells of ataxic mutant mouse stargazer. J. Neurosci..

[50] Chen L., Chetkovich D.M., Petralia R.S., Sweeney N.T., Kawasaki Y., Wenthold R.J., Bredt D.S., Nicoll R.A. (2000). Stargazin regulates synaptic targeting of AMPA receptors by two distinct mechanisms. Nature.

[51] Tomita S., Chen L., Kawasaki Y., Petralia R.S., Wenthold R.J., Nicoll R.A., Bredt D.S. (2003). Functional studies and distribution define a family of transmembrane AMPA receptor regulatory proteins. J. Cell Biol..

[52] Tomita S., Adesnik H., Sekiguchi M., Zhang W., Wada K., Howe J.R., Nicoll R.A., Bredt D.S. (2005). Stargazin modulates AMPA receptor gating and trafficking by distinct domains. Nature.

[53] Kato A.S., Zhou W., Milstein A.D., Knierman M.D., Siuda E.R., Dotzlaf J.E., Yu H., Hale J.E., Nisenbaum E.S., Nicoll R.A. (2007). New transmembrane AMPA receptor regulatory protein isoform, gamma-7, differentially regulates AMPA receptors. J. Neurosci..

[54] Kato A.S., Siuda E.R., Nisenbaum E.S., Bredt D.S. (2008). AMPA receptor subunit-specific regulation by a distinct family of type II TARPs. Neuron.

[55] Sumioka A., Brown T.E., Kato A.S., Bredt D.S., Kauer J.A., Tomita S. (2011). PDZ binding of TARPγ-8 controls synaptic transmission but not synaptic plasticity. Nat. Neurosci..

[56] Schwenk J., Harmel N., Zolles G., Bildl W., Kulik A., Heimrich B., Chisaka O., Jonas P., Schulte U., Fakler B. (2009). Functional proteomics identify cornichon proteins as auxiliary subunits of AMPA receptors. Science.

[57] Kato A.S., Gill M.B., Ho M.T., Yu H., Tu Y., Siuda E.R., Wang H., Qian Y.W., Nisenbaum E.S., Tomita S. (2010). Hippocampal AMPA receptor gating controlled by both TARP and cornichon proteins. Neuron.

[58] Schwenk J., Harmel N., Brechet A., Zolles G., Berkefeld H., Müller C.S., Bildl W., Baehrens D., Hüber B., Kulik A. (2012). High-resolution proteomics unravel architecture and molecular diversity of native AMPA receptor complexes. Neuron.

[59] Shanks N.F., Savas J.N., Maruo T., Cais O., Hirao A., Oe S., Ghosh A., Noda Y., Greger I.H., Yates J.R. (2012). Differences in AMPA and kainate receptor interactomes facilitate identification of AMPA receptor auxiliary subunit GSG1L. Cell Rep..

